# Enhancing the Efficacy of CAR-T Cell Production Using BX795 and Rosuvastatin in a Serum-Free Medium

**DOI:** 10.3390/ijms26072988

**Published:** 2025-03-25

**Authors:** Abed Al-Kader Yassin, Rajashri Banerji, Baisali Bhattacharya, Olga Radinsky, Uzi Hadad, Bar Kaufman, Angel Porgador

**Affiliations:** 1 The Shraga Segal Department of Microbiology, Immunology and Genetics, Faculty of Health Sciences, Ben-Gurion University of the Negev, Beer-Sheva 8410501, Israel; yassinab@post.bgu.ac.il (A.A.-K.Y.); rajashri@bgu.ac.il (R.B.); baisali@post.bgu.ac.il (B.B.); kisterev@post.bgu.ac.il (O.R.); barkau@post.bgu.ac.il (B.K.); 2 The Ilse Katz Institute for Nanoscale Science and Technology, Marcus Campus, Ben-Gurion University of the Negev, Beer-Sheva 8410501, Israel; uzihad@bgu.ac.il

**Keywords:** CAR-T, lentivirus, Nutri-T, PBMCs, BX795, rosuvastatin

## Abstract

Chimeric Antigen Receptor T-cell (CAR-T) therapy has emerged as a transformative approach for cancer treatment, demonstrating remarkable success in patients with relapsed and refractory hematological malignancies. However, challenges persist in optimizing CAR-T cell production and improving therapeutic outcomes. One of the major hurdles is the efficiency of retroviral or lentiviral transduction during CAR-T cell manufacturing. Additionally, the heterogeneity of T-cell populations isolated from patients can impact CAR-T cell effectiveness and persistence in vivo. This article explores a novel strategy to address these challenges by focusing on serum-free medium and additive optimization. We propose a unique approach that incorporates the culturing of T cells in Nutri-T medium, along with 24 h of exposure to combined low concentrations of BX795 and rosuvastatin, to enhance the transduction efficacy and functionality of CAR-T cells. The results presented here provide promising insights into the potential of this strategy to produce more effective CAR-T cells for immunotherapy, ultimately advancing the field and benefiting cancer patients worldwide.

## 1. Introduction

CAR T-cell therapy is a groundbreaking immunotherapeutic approach that has revolutionized cancer treatment. It entails altering a patient’s own T cells genetically so that they express chimeric antigen receptors (CARs) that can identify and attack cancer cells. CARs are created to bind specific antigens on the surface of cancer cells, ultimately causing those cells to die [1]. High response rates and sustained remissions have been achieved with this treatment in patients with relapsed and refractory acute lymphoblastic leukemia (ALL) and lymphoma. The success of CAR-T-cell treatment has resulted in the recent approval of several commercial CAR-T products [2]. These products have proven superior to conventional therapy methods, increasing survival rates and decreasing disease burden [3]. However, issues remain unresolved with respect to improving CAR-T cells’ therapeutic effectiveness and reducing related toxicity [4]. One of the primary problems with CAR-T manufacturing is the pace at which retroviruses or lentiviruses transduce cells. Isolating and cultivating T cells from patients can potentially result in developing a heterogeneous population with variable degrees of activation and differentiation statuses. This variability may impact CAR-T cells’ efficiency and persistence in vivo. Consistent clinical outcomes depend on quality improvement techniques and consistent CAR-T cell manufacturing.

Serum-free media have been highlighted as crucial for enhancing CAR-T cell production procedures, accelerating the time to market, and expanding patient access to these treatments [5]. Additionally, it emphasizes how serum-free media can resolve obstacles, restrictions, and expenses related to serum-containing media. A study comparing AIM-V media with TexMACS media showed that TexMACS stimulates the proliferation of the T effector cell phenotype. At the same time, AIM-V promotes the expansion of the T central memory cell phenotype. Additionally, the expression of T cell exhaustion markers such as Tim3 was found to be elevated [6]. Similarly, in a comparative analysis of activation markers CD69 and CD25 in CD4 and CD8 T cells, a human-plasma-like medium enhanced T cell activation and is more effective than RPMI [7]. Furthermore, an investigation into a chemically defined basal medium (1B2H) developed for T cell expansion revealed that it expanded both naïve and CCR7 levels in T cells equally regardless of the presence or absence of 5% human serum [8].

Moreover, various enhancers have been used with serum-free medium to transduce viruses into T cells successfully [9,10,11]. It has been reported that vectofusin, prostaglandin E29, and dextran improve the lentiviral vector transduction of hematopoietic stem cells, progenitor cells, and mesenchymal stromal cells, respectively [12,13,14]. Additionally, the low-density lipoprotein (LDL) receptor on lymphocytes may be upregulated, enhancing vesicular stomatitis virus-glycoprotein (VSV-G) lentiviral transduction [15]. However, they have drawbacks, such as a small genetic cargo capacity [16]. Among these, rosuvastatin is a drug in the statin class, which is frequently prescribed to treat hypercholesterolemia and cardiovascular disorders. It has been thoroughly investigated in clinical studies and is well known for its powerful lipid-lowering properties [17]. According to a study, rosuvastatin and geranylgeranyl–pyrophosphate combined most effectively increased viral transduction without reducing the NK cells’ capacity to cause cytotoxicity [18]. Rosuvastatin enhances lentiviral transduction efficiency in NK cells by upregulating low-density lipoprotein receptors (LDLRs), facilitating increased viral entry and more effective genetic modification. In addition to its role in transduction, rosuvastatin exerts anti-inflammatory effects, potentially creating a more favorable immune microenvironment for adoptive cell therapies. It mitigates T cell activation and exhaustion, downregulating key markers such as PD-1 and Tim3, which are associated with CAR-T cell dysfunction and reduced therapeutic efficacy [19]. This suggests a potential role in promoting CAR-T cell persistence and functional longevity. Furthermore, rosuvastatin activates the JAK2-STAT3 signaling pathway and contributes to therapeutic angiogenesis, suggesting additional mechanisms that may enhance immune cell function, survival, and persistence within the tumor microenvironment [20]. Clinical evidence further supports the potential benefits of statin use in CAR-T cell therapy, with studies indicating that rosuvastatin administration is associated with improved progression-free survival and overall survival in patients undergoing CD19-CAR-T therapy for aggressive B-cell lymphomas [21]. These findings underscore the relevance of rosuvastatin as a potential adjunct in next-generation CAR-T and NK cell therapies, warranting further investigation into its mechanistic contributions to cellular immunotherapy.

Similarly, BX795, a pharmacological inhibitor of the TBK1/IKKɛ complex, has been shown to enhance lentiviral transduction in human NK cells [19,20]. Additionally, studies have reported its role in improving transduction efficiency in both NK and T cells [21]. As a TBK1/IKKɛ inhibitor, BX795 overcomes the natural resistance of NK cells to viral transduction by temporarily suppressing antiviral defenses, thereby facilitating more efficient genetic modification without compromising cell viability, function, or proliferation [22]. This improved transduction efficiency results in higher CAR expression, which may enhance the anti-tumor activity of engineered cells. Furthermore, BX795 treatment has been reported to increase the proportion of CD8+ T cells within transduced populations, a key advantage for strengthening cytotoxic responses and promoting durable anti-tumor immunity [23]. Its ability to promote the transduction of large-payload lentiviral vectors also enables the delivery of complex genetic circuits or multiple CAR constructs, expanding therapeutic possibilities.

A serum-free Nutri-T medium has yielded reliable and consistent outcomes for T cells generated by patients and healthy donors. The Ella Lemelbaum Institute for Immuno-Oncology at Sheba Medical Center in Israel created the xeno-free Nutri-T formulation, which functions even at low seeding concentrations as low as 15,000 cells [24]. This article provides a unique strategy for increasing the viral transduction rate during CAR-T cell production along with increased functionality in Nutri-T while incorporating statin (rosuvastatin) and a multi-kinase inhibitor (BX795). This process will aid in producing effective CAR-T cells for immunotherapy.

## 2. Results

### 2.1. Proliferation and Central Memory Phenotype of T Cells in Different Media

**Figure 1:**
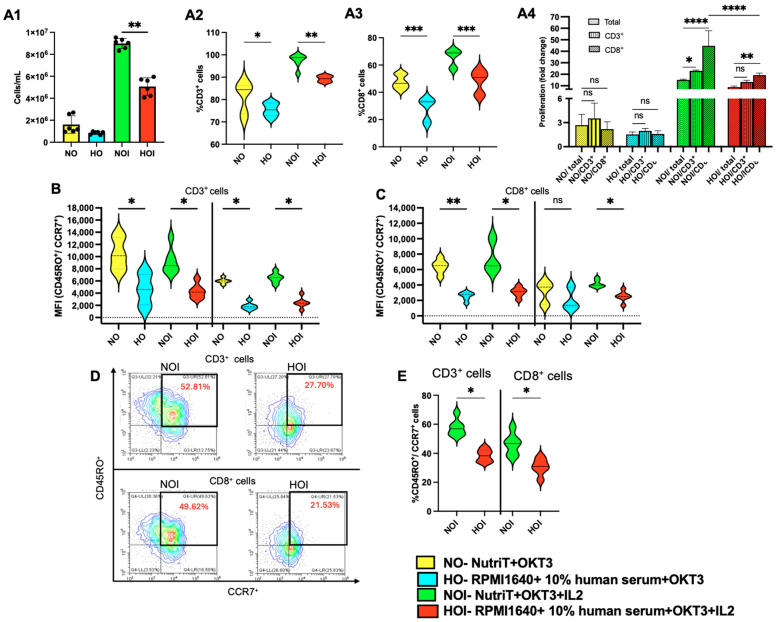
Proliferation and central memory phenotype of non-infected T cells in different media. (**A1**) In total, 600,000 PBMCs were plated in a 48-well plate after isolation and cultured in different media (NO, HO, NOI, and HOI). The cells were counted on day 7. (**A2**) Percentage of CD3-positive cells on day 7 after culturing in different media (NO, HO, NOI, and HOI). (**A3**) Percentage of CD8-positive cells on day 7 after culturing in different media (NO, HO, NOI, and HOI). (**A4**) Proliferation fold change on day 7 for CD3+ and CD8+ cells. Fold change was calculated based on the proliferation fold of total cells and the fraction ratio of CD3 and CD8 cells on day 1 and day 7. Two-way ANOVA was performed using GraphPad Prism 10. (**B**) MFI of CD45RO^+^/CCR7^+^ cells selected from CD3^+^ T cells on day 7 for central memory after culturing the cells in different media. (**C**) MFI of CD45RO^+^/CCR7^+^ cells selected from CD8^+^ T cells on day 7 for central memory after culturing cells in different media. (**D**) Representative plots for CD45RO^+^CCR7^+^ from CD3^+^ and CD8^+^ T cells cultured in NOI and HOI, respectively. (**E**) Percentage of double positive cells for CD45RO and CCR7 from CD3^+^ and CD8^+^ cells cultured in NOI and HOI. To check the markers for memory in CD3^+^ and CD8^+^ T cells cultured in different media, 50,000 cells were stained with 1 ng/mL of APC-conjugated anti-human CD45RO (UCHL1) and PE-conjugated anti-human CCR7 (G043H7) for 30 min. In total, 1 μg/mL of DAPI was used to stain dead cells. All samples were analyzed using a Beckman CytoFLEX L flow cytometer, CA, USA. All experiments were performed with three donors in duplicates. Mann–Whitney and Kruskal–Wallis tests were performed using GraphPad Prism. Asterisks represent significant differences (ns: non-significant; *p* < 0.05 *; *p* < 0.01 **; *p* < 0.001 ***, *p* < 0.0001 ****).

To determine the most favorable condition for transducing T cells, we initially performed a comparison study of culturing non-transduced T cells between different media: 100 ng/mL OKT3-activated cultured cells in Nutri-T (NO), 100 ng/mL OKT3-activated cultured cells in RPMI 1640 + 10% human serum (HO), NO supplemented with 200 U IL2 (NOI), and HO supplemented with 200 U IL2 (HOI). Since a study depicted that RPMI 1640 needed the addition of human serum to promote T cell proliferation, we cultured the T cells with 10% human serum in RPMI 1640 [8]. While Nutri-T is a serum-free medium, we checked whether it affected the proliferation rate of T cells. After PBMC isolation, cells were cultured in NO, HO, NOI, and HOI, and they were counted on day 7 to assess proliferation. It was observed that NOI significantly increased (*p* < 0.01) the proliferation of total cells compared to HOI (Figure 1(A1) for total cell number and Figure 1(A4) for proliferation fold compared to the 6 × 10^5^/well PBMCs seeded on day 1). A significant increase (*p* < 0.05/*p* < 0.01/*p* < 0.001) in the fraction of CD3-positive and CD8-positive cells on day 7 was observed when cultured in IL-2-supplemented NutriT media (Figure 1(A2,A3)). Consequently, the proliferation fold of CD3- and CD8-positive cells could be calculated based on the proliferation fold of total cells and the fraction ratio of CD3 and CD8 cells on day 1 and day 7 (following culture with specific medium conditions). Figure 1(A4) also shows the proliferation fold of CD3- and CD8-positive cells; a significant increase in the proliferation fold of CD3-positive and CD8-positive cells was observed on day 7 when cultured in NOI. Moreover, staining for CD45RO and CCR7 from CD3-positive cells (Figure 1B) and CD8-positive cells (Figure 1C) on day 7 was performed to analyze whether the media affected the central memory of the T cells. Figure 1D depicts the representative plots for CD45RO^+^CCR7^+^ in CD3-positive and CD8-positive cells. Likewise, the increased expression (*p* < 0.05) of CD45RO and CCR7 was observed in CD3-positive and CD8-positive cells when cultured in NOI (Figure 1E). Figure S1 depicts the gating strategy employed to analyze the CD3-positive cells. Thus, it was concluded that Nutri-T significantly increased (*p* < 0.05/*p* < 0.01) the central memory subset of T cells compared to RPMI 1640 in all variations.

Additionally, flow cytometry data indicated that T cell activation leads to an increased cell volume on day 1, with the largest volume observed in NOI. While the cell volume decreased by day 7, NOI still exhibited the highest volume (Figure S2). Thus, NOI significantly increased the cell proliferation and functional memory of non-infected T cells compared to HOI.

### 2.2. Activation and Exhaustion of T Cells in Different Media

**Figure 2:**
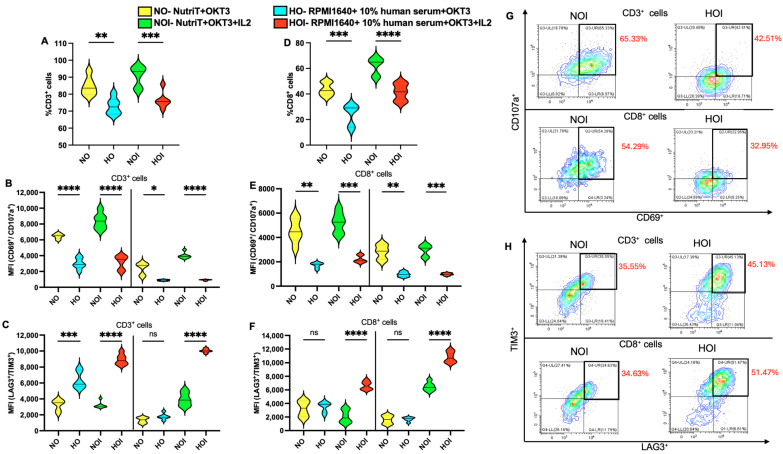
Activation and exhaustion of non-infected T cells in different media. (**A**) Percentage of CD3^+^ cells cultured in different media on day 3. (**B**) MFI of CD69^+^/CD107a^+^ cells selected from CD3^+^ T cells on day 1 for activation. (**C**) MFI of LAG3^+^/TIM^+^ cells selected from CD3^+^ T cells on day 7 for exhaustion. (**D**) Percentage of CD8^+^ cells on day 7. (**E**) Geomean of CD69^+^/CD107a^+^ cells selected from CD8^+^ T cells on day 1 for activation. (**F**) MFI of LAG3^+^/TIM^+^ cells selected from CD8^+^ T cells on day 7 for exhaustion. (**G**) Representative plots for CD107a^+^CD69^+^ from CD3^+^ and CD8^+^ T cells cultured in NOI and HOI, respectively. (**H**) Representative plots for TIM3+LAG3+ from CD3^+^ and CD8^+^ T cells cultured in NOI and HOI, respectively. In total, 50,000 cells were plated in a 96-well plate. For activation, cells were incubated with PE-conjugated anti-human CD3 (UCHT1), AF750-conjugated anti-human CD8 (HIT8a), Perc5.5-conjugated anti-human CD69 (FN50), and APC-conjugated anti-human CD107a (H4A3) for 30 min on ice. For exhaustion, cells were incubated with PE-conjugated anti-human CD3 (UCHT1), AF750-conjugated anti-human CD8, Perc5.5-conjugated anti-human LAG3 (11C3C65), and APC-conjugated anti-human TIM3 (A18087E) for 30 min on ice. All antibodies were used at a concentration of 1 ng/mL. In total, 1 μg/mL of DAPI was used to stain dead cells. All samples were analyzed in the Beckman CytoFLEX L flow cytometer. All experiments were performed with three donors in duplicates. One-way ANOVA test was performed for (**A**,**D**), and Brown–Forsythe and Welch ANOVA tests were performed for (**B**,**C**,**E**,**F**) due to significantly different SDs; statistical analysis was performed using GraphPad Prism. Asterisks represent significant differences (ns: non-significant; *p* < 0.05 *; *p* < 0.01 **; *p* < 0.001 ***; *p* < 0.0001 ****).

To check if the media influenced the activation and exhaustion of T cells, we analyzed the expression of CD69/CD107a and TIM3/LAG3, respectively, through flow cytometry. It was observed that the percentage of CD3-positive cells on day 3 increased significantly (*p* < 0.01/*p* < 0.001) when cultured in Nutri-T compared to RPMI 1640 in all variations (Figure 2A). Also, activation markers such as CD69 and CD107a from CD3-positive cells on day 1 increased significantly (*p* < 0.05 to *p* < 0.0001) in Nutri-T compared to RPMI 1640 in all variations (Figure 2B). Thus, basal activation is higher in Nutri-T. Next, we analyzed the exhaustion status of T cells. LAG3 and TIM3 exhaustion markers observed on day 7 in CD3-positive cells increased significantly (*p* < 0.0001) in HOI compared to NOI (Figure 2C).

Additionally, the percentage of CD8-positive cells on day 7 increased significantly (*p* < 0.001/*p* < 0.0001) when cultured in Nutri-T compared to RPMI 1640 in all variations (Figure 2D). A similar pattern was observed upon assessing the activation markers (Figure 2E) on day 1. On the other hand, the exhaustion markers on day 7 were significantly higher for CD8-positive T cells cultured in HOI compared to NOI (Figure 2F). Figure 2G depicts the representative plots for CD107a^+^CD69^+^ in CD3-positive and CD8-positive cells. Figure 2H depicts representative plots for TIM3^+^LAG3^+^ in CD3-positive cells and CD8-positive cells. Therefore, NOI significantly enhanced the percentage of CD3^+^ and CD8^+^ cells. Moreover, non-infected T cells depicted a high expression of activation markers and a low expression of exhaustion markers when cultured in NOI compared to HOI.

### 2.3. Effect of Nutri-T on Transduction Rate of CAR-HER2-EGFP Viruses Relative to T Cells

**Figure 3:**
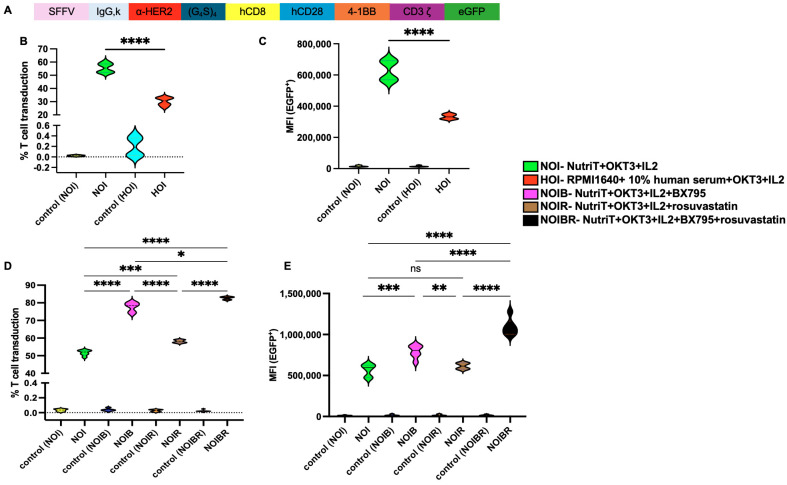
T cell transduction with CAR-HER2-EGFP in human serum and Nutri-T media. (**A**) Schematic representation of third-generation CAR-HER2-EGFP consisting of an SFFV promoter with α-HER2 sequences situated between the adapter and linker, followed by the co-stimulatory domain consisting of CD28 and 4-1BB, along with CD3ζ and EGFP as the fluorescent reporters. (**B**) Transduction percentage of CAR-HER2-EGFP in T cells when cultured in NOI or HOI. (**C**) MFI of T cell transduction when cultured in NOI or HOI. (**D**) Transduction percentage of CAR-HER2-EGFP in T cells when cultured in NOI, NOIB, NOIR, and NOIBR. (**E**) MFI of T cell transduction when cultured in NOI, NOIB, NOIR, and NOIBR. In total, 600,000 T cells cultured in different media were transduced with CAR-HER2-EGFP viruses. Different culture conditions were maintained, and EGFP+ cells were analyzed via a Beckman CytoFLEX L flow cytometer after staining the cells with PE-conjugated anti-human CD3 (UCHT1), followed by DAPI staining. Activated (with 100 ng/mL of OKT3) yet non-infected T cells cultured in their respective media were used as controls. All experiments were performed with two donors in triplicate. One-way ANOVA test was performed for (**B**,**C**,**E**), and Brown–Forsythe and Welch ANOVA tests were performed for (**D**) due to significantly different SDs; statistical analysis was performed using GraphPad Prism. Asterisks represent significant differences (ns: non-significant; *p* < 0.05 *; *p* < 0.01 **; *p* < 0.001 ***; *p* < 0.0001 ****).

After establishing that T cells respond better in Nutri-T media, we checked if it affected the transduction efficiency in T cells. Thus, we first performed lentivirus titration to check the efficacy of the virus encoding EGFP. The transduction units (TUs) of the lentivirus, which represent the number of functional viral particles in a solution that can transduce a cell and express the transgene, were calculated. Figure S3 depicts that increasing the dilution is directly proportional to the transduction units/mL. Thus, a higher number of particles is required for a successful transduction. A third-generation CAR-HER2-EGFP construct is described in Figure 3A.

The PBMCs that were isolated were activated with 100 ng/mL of anti-human CD3 (OKT3), transduced with CAR-HER2-EGFP viruses, and subjected to culturing them in their respective media: Nutri-T-IL-2 (NOI) or RPMI1640+ 10% human serum-IL-2 (HOI). The transduction percentage (Figure 3B) and MFI (Figure 3C) significantly increased (*p* < 0.01) when CAR-HER2-EGFP cells were cultured in NOI. Non-infected T cells cultured in respective media were used as controls.

Then, we aimed to check the effect of BX795 (6 nM) and rosuvastatin (5 nM) on the transduction efficacy of T cells using the following combinations in Nutri-T media: NOI supplemented with BX795 (NOIB), NOI supplemented with rosuvastatin (NOIR), and NOI supplemented with BX795 and rosuvastatin (NOIBR). Culture in NOI medium resulted in an average percent transduction of 51.7%; when BX795 was added as a single treatment (NOIB), the transduction percentage significantly increased to an average of 77.7%. Also, rosuvastatin (NOIR) as a single treatment significantly enhanced the average percent transduction to 58.1 (as compared to NOI). Upon adding BX795 and rosuvastatin together, the average percent transduction increased in NOIBR to 82.7%, which was a significant enhancement as compared to either NOIB or NOIR (*p* < 0.05 or *p* < 0.0001, respectively; Figure 3D). Similar yet more prominent results were observed upon analyzing the geomean data of transduced EGFP; the MFI of NOIBR was significantly higher than either NOIB or NOIR (Figure 3E). Importantly, it is to be noted that the concentrations for BX795 or single rosuvastatin treatments reported to date ranged from 2 to 10 µM [21] and 0.5 to 20 µM [18], respectively. However, our method effectively promotes the transduction rate when these two enhancers are used at low concentrations of 5–6 nM, which are less toxic to the treated T cells [8,18,23,24,25]. Thus, NOIBR was found to be the most effective media for achieving higher transduction efficiencies for CAR-HER2-EGFP.

### 2.4. Evaluation of CAR-HER2-EGFP for Functional Activity

**Figure 4:**
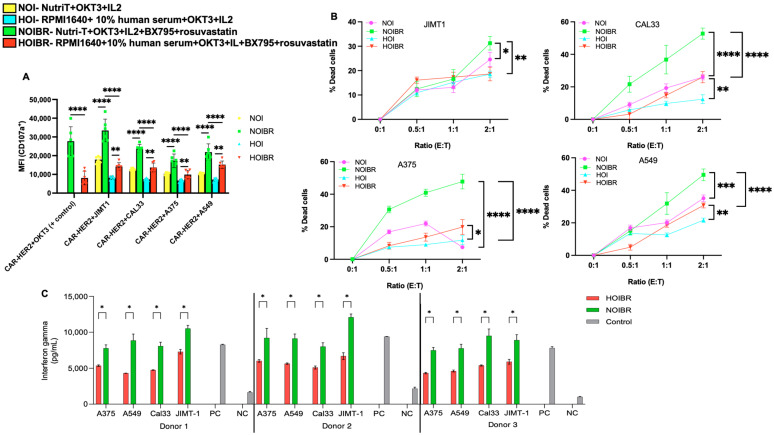
Functional assays for CAR-HER2-EGFP-positive T cells. (**A**) Degranulation assay. One hundred-fifty thousand target cells/well and fifty thousand CAR-HER2-EGFP cells/well (previously cultured in NOIBR or HOIBR) were plated in 96-well plates, along with PC5.5-conjugated anti-human CD107a. The cells were incubated for 3 h at 37 °C with 5% CO_2_. The cells were washed and stained with PC-C5.5-conjugated anti-human CD107a for 30 min on ice. Flow cytometry was performed to evaluate degranulation. CAR-HER2-EGFP alone was used as a negative control. Plastic-coated 1 μg/mL of anti-human CD3 (OKT3) for 30 min was used as a positive control. Data are normalized to negative controls. (**B**) Killing assay. Twenty thousand DiD-labeled JIMT1s were incubated with different ratios of sorted CAR-HER2-EGFP (effector: target/E:T) cultured in different media for 3 h at 37 °C with 5% CO_2_. The cells were washed and stained with 1 µg/mL of DAPI, followed by flow cytometry analysis. JIMT1 cells labeled with DiD were only used as a negative control. All experiments were performed with two donors in triplicate. Multiple-T tests were performed using GraphPad Prism. (**C**) IFNγ secretion. A hundred thousand cells of each cell line were placed in a 96-well flat-bottom plate and incubated for 24 h with twenty thousand PBMCs isolated from 3 donors at a 1:5 ratio. The incubation took place in NOIBR and HOIBR. Following incubation, the supernatant was collected from the wells and analyzed using a standard IFminN-γ ELISA assay. A two-way ANOVA test was performed. Asterisks represent significant differences (*p* < 0.05 *; *p* < 0.01 **; *p* < 0.001 ***; *p* < 0.0001 ****).

To analyze whether the constructed CAR-HER2-EGFP is functional in NOIBR media, we performed a degranulation assay on day 7 by quantifying the surface CD107a marker on CAR-HER2-EGFP when incubated with the JIMT1, CAL33, A375, and A549 target cell through flow cytometry. CAR-HER2-EGFP cells alone were used as a negative control for degranulation, while plastic-coated anti-human CD3 (OKT3) was used as a positive control. From Figure 4A, it was observed that the degranulation of CAR-HER2-EGFP was highly significant (*p* < 0.05) when the cells were cultured in NOIBR and incubated with target cell lines compared to HOIBR, indicating the higher functionality of the constructed CAR-T cells in NOIBR media. Figure S4 depicts the representative plots for degranulation assays when T cells were cultured in different media.

Next, we performed a killing assay against JIMT1, CAL33, A375, and A549 cells using NOIBR and HOIBR. Figure 4B depicts the percentage of dead target cells due to CAR-HER2-EGFP cells, and S5 depicts the representative plots. It can be observed that T cells cultured in NOIBR were significantly more effective than HOIBR (*p* < 0.05 to *p* < 0.0001) at a 2:1. Figure 4C represents IFNγ secretion after 24 h for three donors, and it can be observed that NOIBR exhibited higher IFNγ secretion than HOIBR (*p* < 0.05), thus indicating the effectiveness of transduced T cells cultured in NOIBR. Hence, CAR-HER2-EGFP cultured in NOIBR showed the highest functionality.

OKT3 binds to CD3 and activates T cells. NutriT enhances the activation and functional memory of T cells. BX795 acts as a TBK1 inhibitor, thus avoiding antiviral immunity. Rosuvastatin increases the LDL receptor, further enhancing the viral transduction efficiency and finally expressing functional CAR-T cells.

## 3. Discussion

It is essential to select an adequate cell culture medium to maintain the health and functionality of the relatively small populations of cells present at the start of the CAR-T cell manufacturing process and following gene transfer. The use of serum-free media presents a potential solution to the difficulties, constraints, and expenses related to serum-containing media, including problems with varying quality, the demand for significant replenishment, and the complexity of regulatory requirements [26]. In this study, we propose using serum-free media, along with statin and Tank-binding Kinase-1 (TBK-1) inhibitors, to increase the transduction rate of T cells for the generation of CAR-T.

A previous study suggested the use of modified buffers along with electroporation, although the highest transfection rate in human T cells was observed to be 41–59% [27]. Meanwhile, IL-21 increased the transduction efficiency of the virus to T cells by 45% on day 12 [28]. Similarly, 72.3% transduction efficiency was observed in murine CD4 T cells when the cells were activated 16–18 h prior to transduction by CD3/CD28 Dynabeads [29]. Another report suggests the use of spinoculation and retronectin, which increased the transduction of T cells to 63.29% [30]. In this study, we developed a protocol with minimum ease, which results in a transduction efficiency of up to 80%.

Our methodology supports the expansion of T cells over a shorter duration (5–7 days) to preserve a higher proportion of central memory T cells. Shorter culture periods have demonstrated enhanced antitumor efficacy in preclinical studies. For instance, CAR T cells harvested after 3–5 days showed superior tumor control compared to those cultured for 9 days in mouse xenograft models [31]. Moreover, shorter culture periods can improve T cell persistence, survival, and self-renewal after reinfusion, promoting better long-term patient outcomes [32]. Limiting culture time helps maintain the effector function of T cells in vivo. Current FDA guidelines mandate additional testing for culture periods exceeding 96 h post-transduction; adopting a culture period of fewer than 5 days can circumvent this costly requirement [33]. Extended ex vivo culture may lead to T cell exhaustion, potentially diminishing therapeutic efficacy upon patient administration [32]. Additionally, CD107a expression, which is crucial for measuring degranulation, is most robust shortly after activation. By adhering to this schedule, researchers can work with T cells in their most responsive state, leading to more accurate and reliable outcomes in degranulation assays [34].

We aimed to develop a simple, fast, and cost-effective method for transducing and maintaining primary T cells. While the conventional method uses CD3/CD28 beads, our findings show that OKT3 stimulation alone is also effective. OKT3 better preserves a less differentiated T cell phenotype, with T cells expanded using OKT3/IL-2, showing a stronger effector memory profile, which is beneficial for tumor-killing [35,36]. Additionally, soluble OKT3 causes fewer antigen-induced cell deaths in CD8 cells and allows for more precise control over stimulation strength, unlike Dynabeads, which provide fixed stimulation and may require extra purification steps due to bead contamination. Although Dynabeads are effective, particularly for CD4^+^ T cells, OKT3 offers advantages, especially for CD8^+^ T cells.

Similarly, IL2 alone allows the more targeted stimulation of T cell subsets, while adding cytokines such as IL7 or IL15 can complicate responses by activating unintended lymphocyte populations. Focusing on a single cytokine simplifies optimization and enhances reproducibility [37,38]. Even at moderate concentrations (50–200 IU/mL), IL-2 can achieve significant T cell expansion without additional cytokines being needed [38].

T cell activation is a dynamic process that begins rapidly after stimulation. Early activation markers, such as CD69, are upregulated within hours, while CD25 expression increases within 24 h of activation [39]. Assessing these activation markers on the very first days can help capture the peak expression of early and intermediate markers, offering a clear view of the initial T cell response [40]. In contrast, T cell exhaustion develops gradually with prolonged antigen exposure. Inhibitory receptors like PD-1, LAG-3, and TIM-3 show increased expression after several days of stimulation, with exhausted T cells typically displaying reduced effector functions and cytokine production by day 7 compared to activated T cells [41]. This timing strategy helps distinguish between truly activated T cells and those transitioning to an exhausted state, providing a comprehensive view of T cell functionality over time. Examining activation markers early and exhaustion markers later offers valuable insights into the complex dynamics of T cell responses to better understand the progression from activation to potential exhaustion in various immunological contexts [42].

However, studies state that BX795 and rosuvastatin can provide toxicity to T and NK cells. In T cells, treatment with 4 μM BX795 has been shown to preserve cell growth and function, while some studies suggest that 6 μM BX795 maintains low toxicity while achieving optimal transduction efficiency in both NK and T cells. Importantly, BX795’s inhibitory effect on antiviral responses is reversible, enabling its use during the transduction process without permanently altering the cells’ innate antiviral mechanisms. By improving transduction rates without compromising cell viability or function, BX795 contributes to the development of more effective and durable cellular immunotherapies [22,23]. Similarly, in NK cells, while an initial suppression of cytotoxicity was observed, rosuvastatin ultimately enhanced viral transduction without compromising cytotoxic function [18]. It has been demonstrated that rosuvastatin exhibits immune-enhancing effects in certain settings, including an increase in NKT cell percentages and a reduction in TIM-3+ NK cells in chronic hepatitis B patients. Additionally, other statins, such as atorvastatin, have been shown to downregulate TIM-3 and PD-1 expression on T cells, further supporting the potential of statins in modulating T cell exhaustion and immune responses [19,43,44,45].

The optimal concentration of rosuvastatin for enhancing lentiviral transduction is 5 μM, as it maximally upregulates LDLR expression in NK cells without compromising cell viability. At this dose, rosuvastatin increases LDLR protein expression by at least threefold in immune cells. While higher concentrations negatively impact cell viability, lower doses are insufficient in inducing LDLR expression effectively [18].

**Figure 5:**
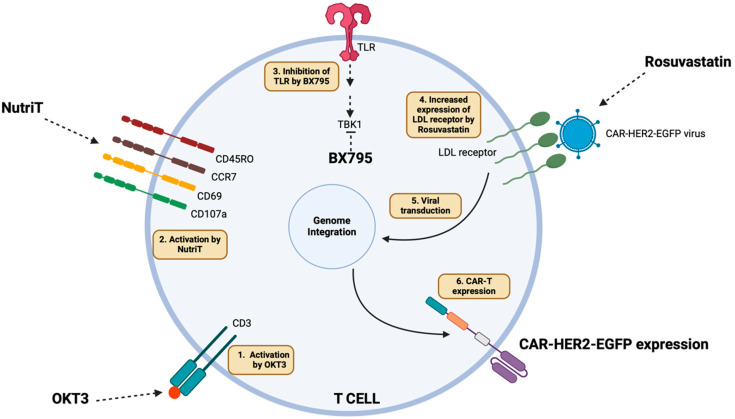
Mechanism of BX795 and rosuvastatin in enhancing CAR-T expression.

In our investigation, Nutri-T by itself improved T cell activation, growth, central memory expansion, and the transduction rate. Rosuvastatin and the TBK-1 inhibitor BX795 were previously reported (used as single treatments) as media additives that could enhance transduction efficacy, yet with plausibly associated toxicity [18,23]. We showed that the addition of both rosuvastatin and BX975 in a serum-free Nutri-T medium in low concentrations and within limited transduction times (24 hrs) significantly enhanced transduction efficacy while retaining functionality. Thus, we believe that NutriT media improve the activation of primary T cells. As a serum-free medium, it avoids exposure to undefined antigens present in serum, which can lead to non-specific activation or anergy. Additionally, many lentiviruses are transduced via the LDL receptor on lymphocytes, enhancing VSV-G lentiviral transduction [15]. Thus, to upregulate the LDL receptor on the surface of the T cells, we used rosuvastatin [17] and BX795, a pharmacological inhibitor of the TBK1/IKKɛ complex (which plays a critical role in the antiviral response) to enhance lentiviral transduction, further improving their cytotoxicity (Figure 5) [22,23,46]. Therefore, this methodology can be utilized to generate stable and efficient CAR-T cells for immunotherapy.

## 4. Methods

### 4.1. Media Preparation and Reagents

HEK293T cells (ATCC CRL-3216) were cultured in DMEM (Gibco, MA, USA) containing 10% Fetal Bovine Serum (FBS) (Gibco, MA, USA), 1% Pen Strep (Gibco, MA, USA), 1mM sodium pyruvate (Sartourious—Beit Haemek, Israel), 0.1 mM NEAA (non-essential amino acids) (Sartourious—Beit Haemek, Israel), 10 mM Hepes (Sartourious—Beit Haemek, Israel), and 2 mM L-glutamine (Sartourious). For culturing PBMCs, RPMI 1640 + 10% human serum (Gibco, MA, USA) and 4Cell^®^ Nutri-T Medium (Sartourious—Beit Haemek, Israel, Cat no. 05-F3F2111-1K) were prepared by supplementing with 200 units/mL of the recombinant cytokine IL-2, Pen Strep, sodium pyruvate, NEAA, Hepes, and L-glutamine. Concentrated stocks of 6 mM BX795 (InVivoGen, CA, USA, Cat no. #tlrl-bx7) and 2.5 mM of rosuvastatin (Crestor, Sigma-Aldrich, Rehovot, Israel, Cat no. 147098-20-2) were prepared by dissolving them in DMSO. When necessary, BX795 and rosuvastatin were added to the media at a final concentration of 6 µM and 5 µM, respectively.

### 4.2. PBMCs Extraction

In total, 10 mL of peripheral blood was extracted from healthy consenting donors using a DG-vein set (VSET21) and placed into an LH Lithium Heparin tube (Greiner Bio-One, Austria, Kremsmünster). The blood was diluted with 1X PBS in a 1:1 ratio, loaded into Ficoll-paqueTM plus (Amersham Bioscience), and separated by centrifugation at 1200× *g* for 30 min. Mononuclear cells were collected at the interphase, washed twice using 1X PBS, and counted, and 600,000 cells/mL were plated on 48-well plates in an RPMI 1640 medium or Nutri-T with 200 U/mL of rhIL2 and 100 ng/mL of anti-human CD3 (OKT3) (BioLegend, San Diego, CA, USA, Cat no. 317347). When required, after 24 h of incubation, the cells were washed twice with 1X PBS, and transduction to T cells was performed.

### 4.3. T Cell Proliferation

Cell proliferation was assessed by counting the number of cells on day 7 after transduction using a DeNovix celldrop automated cell counter, CA, USA. The total number of cells was calculated using the following formula:

Total no. of cells/mL = No. of cells × dilution factor

### 4.4. Activation, Exhaustion, and Memory of T Cells

In total, 50,000 T cells were seeded in a 96-well plate and stained for activation (CD69/CD107a) and exhaustion (Tim3/Lag3). For activation, cells were incubated with PE-conjugated anti-human CD3 (UCHT1) (Cat no. 300441), AF750-conjugated anti-human CD8 (HIT8a) (Cat no. 300932), Perc5.5-conjugated anti-human CD69 (FN50) (Cat no. 310926), and APC-conjugated anti-human CD107a (H4A3) (Cat no. 328620) for 30 min on ice. For exhaustion, cells were incubated with PE-conjugated anti-human CD3 (UCHT1), AF750-conjugated anti-human CD8, Perc5.5-conjugated anti-human LAG3 (11C3C65) (Cat no. 369312), and APC-conjugated anti-human TIM3 (A18087E) (364804) for 30 min on ice. To check the T cell memory function, the cells were stained with APC-conjugated anti-human CD45RO (UCHL1) (Cat no. 304210) and PE-conjugated anti-human CCR7 (G043H7) (Cat no. 353204). All antibodies were used from Biolegend, San Diego, CA, at a 1 ng/mL concentration. The cells were washed with 1X PAF (1% PBS, 0.05% sodium azide, and 1% FBS), and DAPI (1 μg/mL) was used to stain dead cells. All samples were analyzed using the Beckman CytoFLEX L flow cytometer.

### 4.5. Lentivirus Preparation and Titration

The pLENTI CAR-HER2-EGFP vector was used, which consisted of an SFFV promoter and anti-HER2 situated between the linker and adapter, followed by the co-stimulatory domain consisting of CD28 and 4-1BB along with CD3ζ and EGFP as the fluorescent reporter. Lentiviruses were prepared according to the manufacturer’s protocol using the PolyJet™ in vitro transfection reagent (SignaGen laboratories, MD, USA, Cat no. SL100688). Briefly, HEK293T cells were grown to ~90 % confluency in 6-well plates. In total, 0.8 μg of pLENTI CAR-HER2-EGFP vector and 1 μg of packaging plasmids (0.5 µg of pLP1, 0.2 µg of pLP2, and 0.3 µg of VSV-G) were transfected to HEK293T using 5.4 μL of PolyJet™ reagent. The medium was changed after 24 h, and the lentiviruses were collected after 2 days. The supernatant containing lentiviruses was centrifuged at 500× *g* for 5 min to remove the dead HEK293T cells. The supernatant was filtered through a 0.45 µm filter, aliquoted, and stored at −80 °C for further use.

The fluorescence titering assay for Lentivirus by addgene was used to perform the titration. In total, 75,000 HEK293T cells/well in DMEM+ 10% FBS were seeded into a 6-well plate. The cells were incubated at 37 °C with 5% CO_2_ overnight. The media were aspirated, and 1.5 mL of serially diluted viruses prepared in DMEM with 10 μg/mL polybrene was added to cells. HEK293T cells with no viruses were used as a reference for cell counting. After 48 h of incubation, the media were replaced with 1 mL of PBS. The fluorescence-positive cells were analyzed via flow cytometry. The transduction units/mL were calculated using the formula;

T= (N × F × D)/VT

where T = titer (TU/mL); N = number of cells transduced; F = fraction of cells with fluorescence; D = dilution factor; VT = transduction volume, mL.

### 4.6. Transduction in T Cells

For transduction, in a 48-well plate, 400 μL of 600,000 cells was infected with 600 μL of viral supernatant (2 vol:3 vol). When required, BX795 and/or rosuvastatin were added to the wells to a final concentration of 6 nM and 5 nM, respectively. The cells were incubated at 37 °C with 5% CO_2_ for 24 h, and the cells were washed twice with 1X PBS to remove BX795 and rosuvastatin. The cells were re-plated in their respective media without BX795 and rosuvastatin. The cells were then maintained in a Nutri-T medium with 200 U/mL of rIL-2.

### 4.7. Flow Cytometry

After transduction, the T cells were analyzed for EGFP positivity using a Beckman CytoFLEX flow cytometer to check the transduction efficiency. In total, 50,000 cells/well were seeded in a 96-well plate and washed with 1X PAF. The cells were stained with 1 ng/mL of PE-conjugated anti-human CD3 (UCHT1) and incubated for 30 min on ice. The cells were washed twice with 1X PAF, and the dead cells were stained with DAPI (1 μg/mL). All samples were analyzed using a Beckman CytoFLEX L flow cytometer. For gating, unstained controls were analyzed, and CD3-positive cells were selected.

### 4.8. Degranulation Assay

A degranulation assay with CAR-HER2-EGFP was carried out as described [25]. Briefly, 150,000 target cells/well (JIMT1 (RRID CVCL_2077), CAL33 (RRID CVCL_1108), A375 (ATCC CRL-1619), and A549 (ATCC CCL-185)) were seeded in a 96-well plate with 50,000 CAR-HER2-EGFP/well cultured previously in either Nutri-T or RPMI 1640 supplemented with 10% human serum, along with PCP-C5.5-conjugated anti-human CD107a (H4A3) (Biolegend, San Diego, CA, USA, Cat no. 328616). The cells were incubated for 4 h at 37 °C with 5% CO_2_. After 4 h, the cells were washed and stained with PC-C5.5-conjugated anti-human CD107a for 30 min on ice. Flow cytometry was performed to evaluate degranulation. CAR-HER2-EGFP alone was used as a negative control, while 1 μg/mL of anti-human CD3 (OKT3) was used as a positive control.

### 4.9. Killing Assay

After the successful transduction of CAR-HER2-EGFP in different media, the sorting of CAR-HER2-EGFP was performed to carry out the killing assay. We initially performed the killing assay with 20,000 JIMT1 cells (Target-T) labeled with VybrantTM DiD cell-labeling solution (Invitrogen) (1:250 dilution), which were incubated in a 96-well plate with different ratios of CAR-HER2-EGFP (Effector-E) cultured in either Nutri-T or RPMI 1640 supplemented with 10% human serum (E:T ratios—0:1, 0.5:1, 1:1, and 2:1). The plate was incubated for 3 h at 37 °C with 5% CO_2_. After 3 h, the cells were washed and stained with DAPI (1 μg/mL). All samples were analyzed in the Beckman CytoFLEX L flow cytometer.

To avoid labeling with the VybrantTM DiD cell-labeling solution for the killing assay, we prepared CAL33, A375, and A549 cell lines expressing mCHERRY. Briefly, 20,000 target cells were incubated with CAR-HER2-EGFP cultured in either Nutri-T or RPMI 1640 supplemented with 10% human serum at different ratios (E:T ratios—0:1, 0.5:1, 1:1, and 2:1) for 3 h at 37 °C with 5% CO_2_. After 3 h, the cells were washed and stained with DAPI (1 μg/mL). All samples were analyzed in the Beckman CytoFLEX L flow cytometer.

### 4.10. Mean Cell Volume

T cells were cultured in different culture media, and the average T cell volumes were measured to determine the mean cell volume using DeNovix and CellDrop FL on day 0, day 1, and day 7.

### 4.11. Interferon Gamma Assay

To evaluate the impact of different donor cells activated and cultured in various media, four cell lines (JIMT1, CAL33, A549, and A375) were exposed to PBMCs from three healthy donors. These PBMCs were modified with Car Her2 lentivirus, treated with Rosuvastatin and BX795, and sorted for EGFP+ cells to ensure fair comparisons. The immune response was measured through interferon-gamma secretion. The experiment involved incubating PBMCs with different cell lines (1:5 ratio) in a 96-well plate for 24 h. Incubation occurred in either NutriT or RPMI 10% HS media, supplemented with sodium pyruvate, L-glutamine, and antibiotics but without IL-2. After incubation, the supernatant was collected and analyzed using an IFN-γ ELISA assay.

### 4.12. Statistical Analysis

Experiments were performed either with 2 donors in experimental triplicates or with 3 donors in experimental duplicates. Kruskal–Wallis and non-parametric Mann–Whitney U tests were performed using GraphPad Prism (GraphPad Software Inc., La Jolla, CA, USA). Error bars represent standard deviations, while asterisks denote statistical significance (*p* < 0.05 *; *p* < 0.01 **; *p* < 0.001 ***; *p* < 0.0001 ****).

## 5. Conclusions

In conclusion, choosing the suitable cell culture medium is essential for preserving cell functionality during the CAR-T cell manufacturing process, particularly after gene transfer. Our study suggests using a serum-free Nutri-T medium combined with both rosuvastatin and BX795 additives to improve the transduction rate of T cells and the potency of produced CAR-T cells. This method has demonstrated a transduction efficiency of up to 80%, in addition to enhancing T-cell activation, growth, central memory phenotype, and transduction rates. Our results indicate that this technique can effectively produce stable and efficient CAR-T cells for immunotherapy, advancing the field and bringing new hope to cancer patients.

## Data Availability

The original contributions presented in this study are included in the article/Supplementary Material. Further inquiries can be directed to the corresponding author.

## Supplementary Materials

The following supporting information can be downloaded at https://www.mdpi.com/article/10.3390/ijms26072988/s1.
