# Supplementary material for: Enhancing the Efficacy of CAR-T Cell Production Using BX795 and Rosuvastatin in a Serum-Free Medium
Source: Int J Mol Sci. 2025 Mar 25;26(7):2988. doi: 10.3390/ijms26072988 (PMC11988885; doi:10.3390/ijms26072988)
Supplement: Supplementary file 1 [file ijms-26-02988-s001.zip › ijms-3516206-supplementary.pdf]

Supplemental figure:

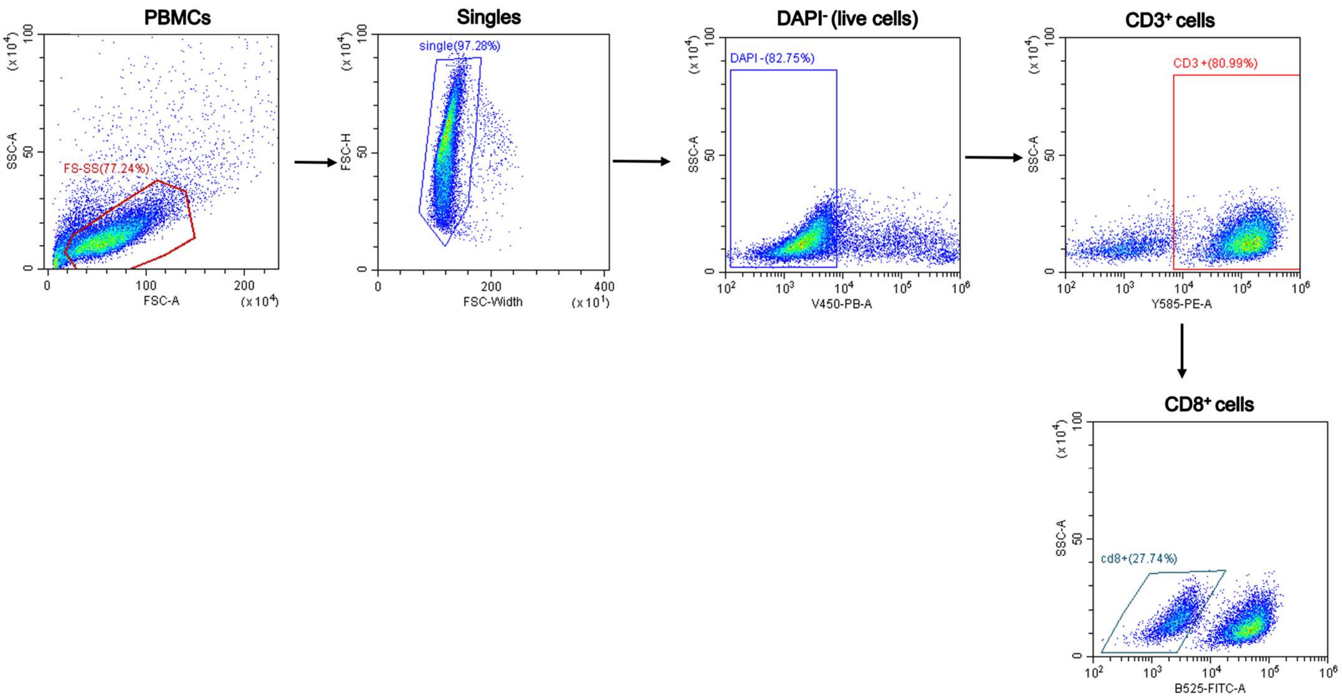

Figure S1. Representative gating strategy for CD3+ cells

Fifty thousand cells/well were seeded in 96 well plate and the T cells were stained with 1 ng/ml of PE-conjugated anti-human CD3 (UCHT1), FITC conjugated anti-human CD8 and incubated for 30 minutes on ice. The cells were washed twice with 1X PAF and the dead cells were stained with DAPI (1 ug/ml). Gating was performed as depicted in the Figure. The PBMCs were selected, followed by gating the singles and DAPI negative. CD3+ cells were then gated and were further gated for FITC for CD8+ cells.

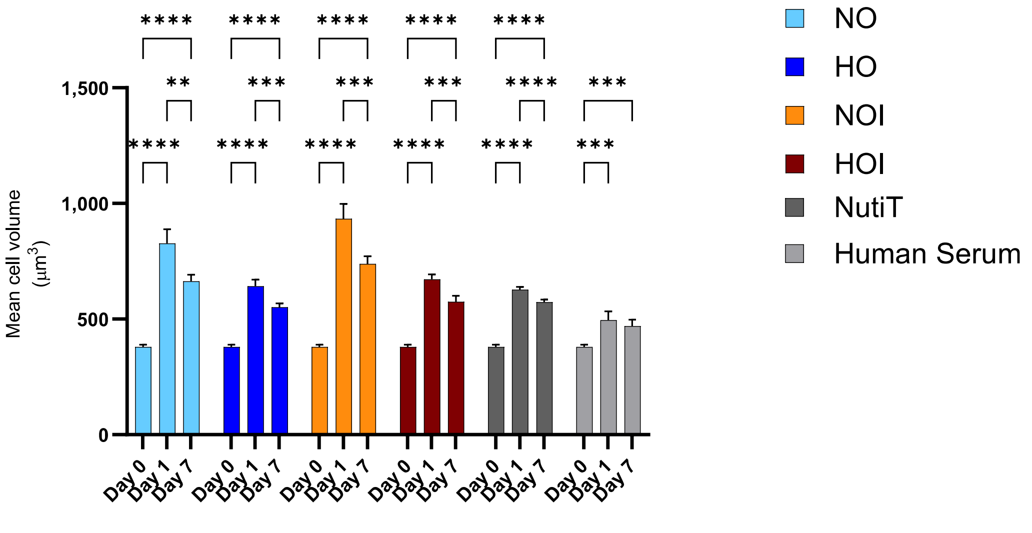

Figure S2. Cell volume in different media

T cell volumes were measured using [DeNovix, CellDrop FL]. T cells volume measured from different conditions through different time points for Day 0,1,7 . (n = [X] mean cell volume µm3, [Y] represent different time points . two-way ANOVA test was performed (ns: non-significant, p <0.0014**, p <0.0004*** , p <0.0001****).

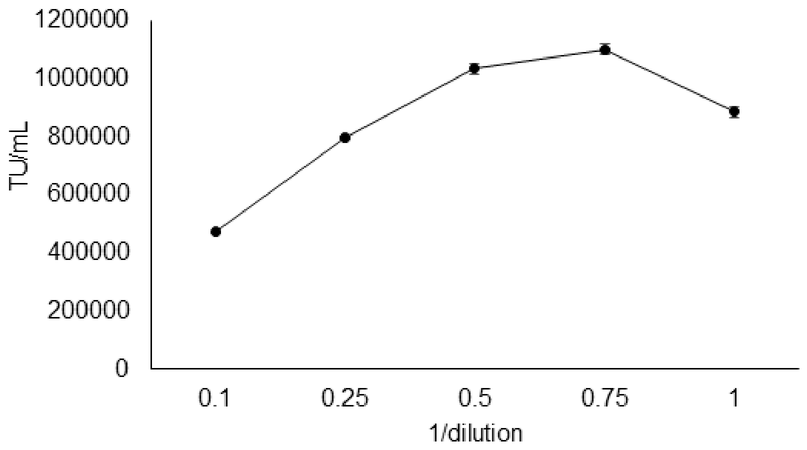

Figure S3. Lentivirus titration curve

Seventy-five thousand HEK293T cells were seeded in 6-well plate and incubated overnight. Serially diluted recombinant lentiviruses encoding GFP were prepared in DMEM with 10 µg/mL polybrene and added to 83,000 cells/well. After 48 h of incubation, the cells were analyzed for GFP expression by flow cytometry.

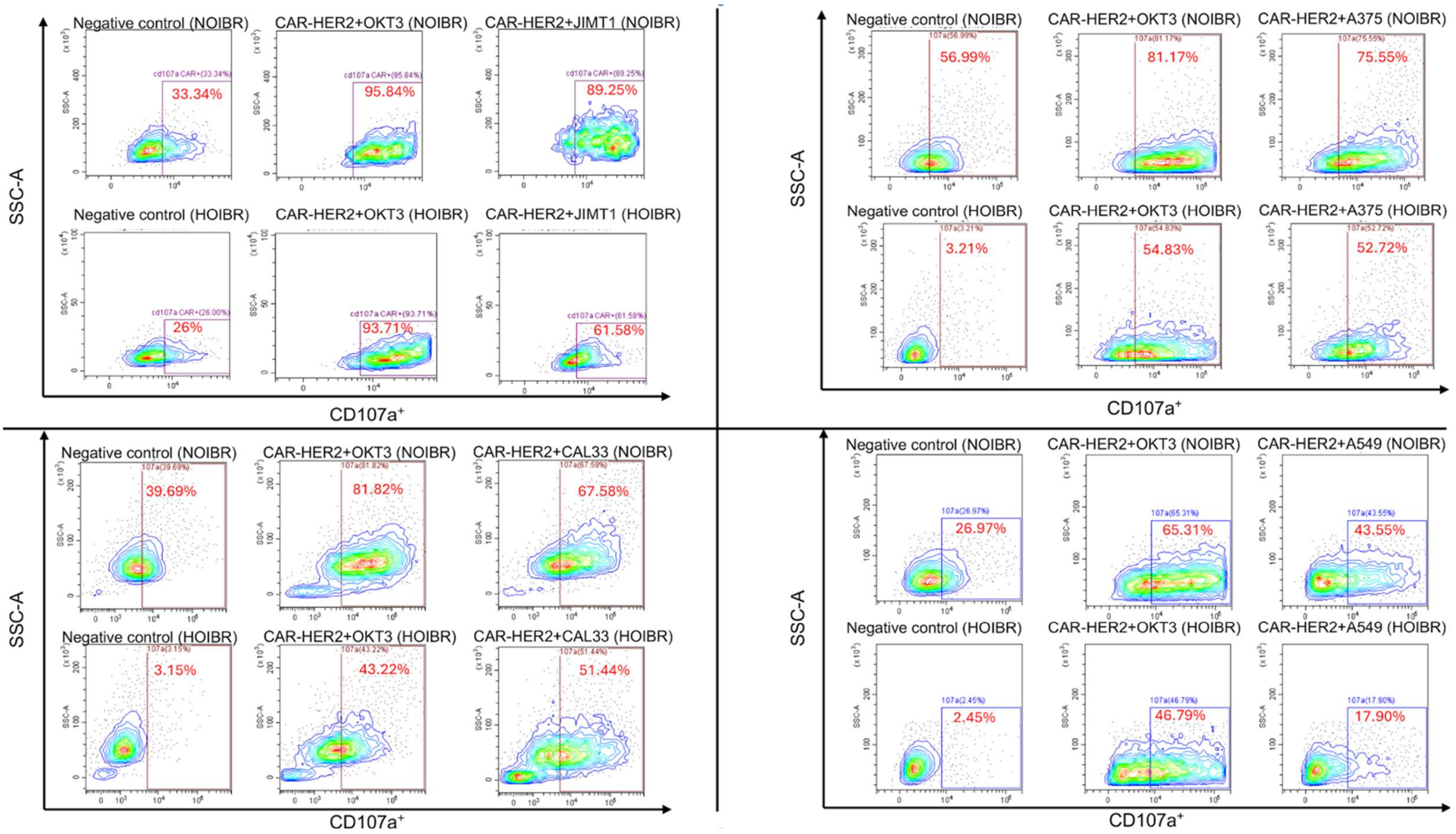

Figure S4. Representative plots for degranulation assay

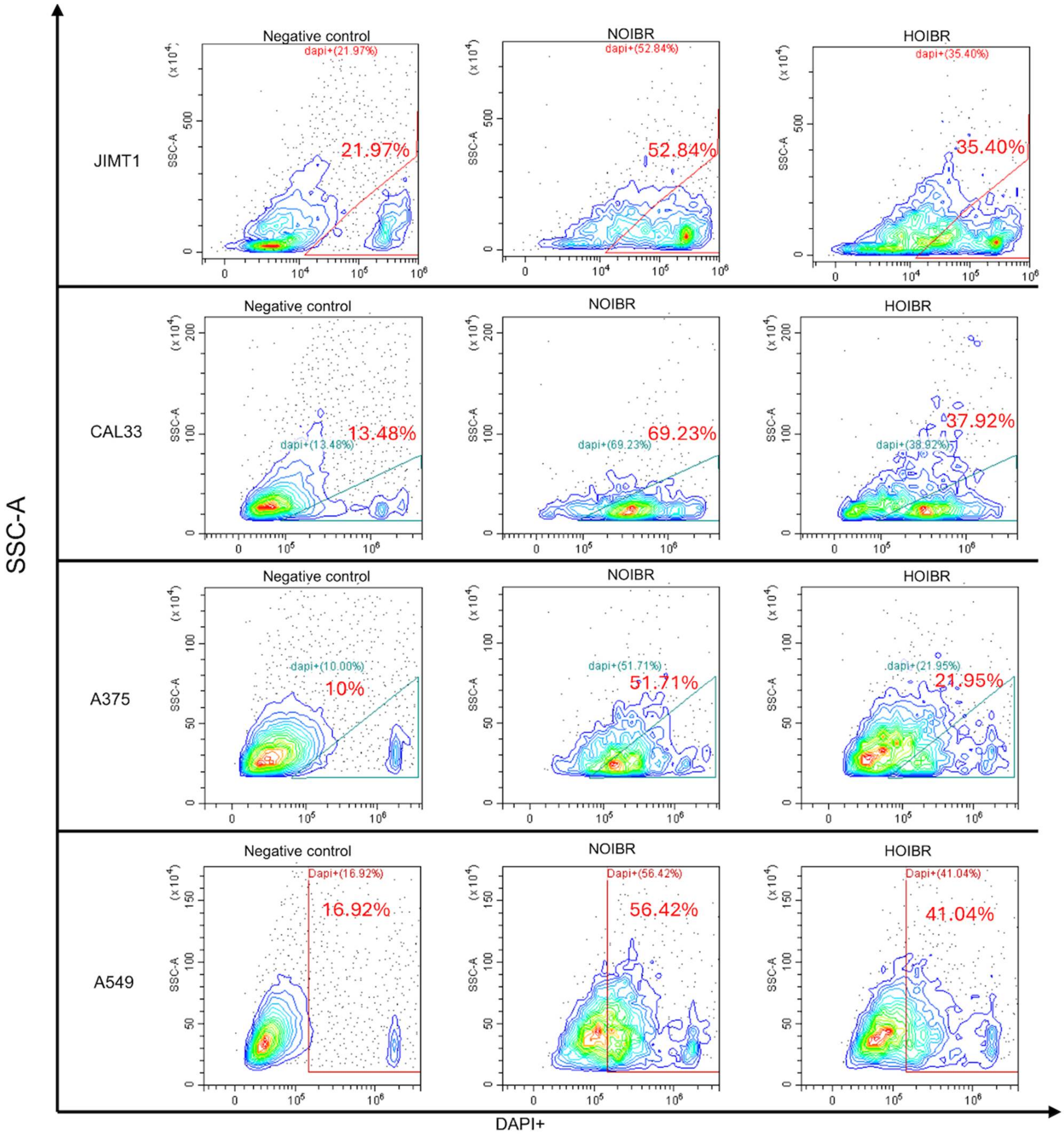

Figure S5. Representative plots for killing assay
